# Antimicrobial resistance crisis in STIs: Gonorrhea and *M. genitalium* on the brink of untreatability

**DOI:** 10.1016/j.nmni.2026.101747

**Published:** 2026-03-29

**Authors:** Nafiseh Ghodsi, Roghayeh Arezoomand, Amir Azimian

**Affiliations:** a Department of Advanced Sciences and Technologies, School of Medicine, North Khorasan University of Medical Sciences, Bojnurd, Iran; b Department of Pathobiology and Laboratory Sciences, Faculty of Medicine, North Khorasan University of Medical Sciences, Bojnurd, Iran

**Keywords:** Sexually transmitted infections, Antimicrobial resistance, *Neisseria gonorrhoeae*, *Mycoplasma genitalium*

## Abstract

**Background:**

Antimicrobial resistance (AMR) in sexually transmitted infections (STIs), especially *Neisseria gonorrhoeae* and *Mycoplasma genitalium*, is a growing global health crisis. These pathogens are developing resistance to first-line treatments like ceftriaxone and macrolides, risking untreatable infections. This review examines resistance trends, treatment challenges, and emerging solutions.

**Methods:**

A systematic analysis of epidemiological data, molecular studies, and clinical trials was performed. Key resistance markers (e.g., *penA* mosaics in *N. gonorrhoeae*, 23S rRNA mutations in *M. genitalium*) and treatment outcomes were assessed. Data sources included WHO reports, peer-reviewed studies (2010–2024), and phase II/III trials of new drugs like zoliflodacin and gepotidacin.

**Results:**

*N. gonorrhoeae* resists all previously effective antibiotics, with ceftriaxone-resistant strains (e.g., FC428 clone) spreading via *penA*-60.001 alleles. Dual resistance to ceftriaxone/azithromycin is emerging. *M. genitalium* shows high macrolide (23S rRNA mutations) and fluoroquinolone (*parC/gyrA* mutations) resistance (>50% in some regions), complicating therapy. While zoliflodacin and gepotidacin show promise, pre-existing resistance (e.g., GyrB D443N) threatens efficacy. Third-line options (pristinamycin, sitafloxacin) have limited availability and effectiveness.

**Discussion:**

The AMR crisis in STIs requires urgent action, including improved surveillance, resistance-guided therapy, and rapid diagnostics. Investment in new antibiotics and alternative approaches (e.g., doxycycline prophylaxis) is crucial. Global public health efforts are needed to curb resistance and prevent untreatable STIs.

## Highlights

• Sexually transmitted infections (STIs) remain a major global public health challenge.
• *M. genitalium* exhibits increasing resistance to antibiotics.
• *N. gonorrhoeae* has progressively acquired resistance to all previously recommended antibiotics.

## 1 Introduction

Sexually transmitted infections (STIs) have afflicted humans throughout history and are documented in numerous historical and medical texts. Clinically, individuals with STIs may present with syndromic symptoms such as vaginal, urethral, or rectal discharge; genital ulcers; or lower abdominal pain. Each syndrome can be caused by various infectious agents. Although understanding and management of STIs have advanced considerably over the past century, some syndromes remain without an identified etiology. STIs significantly impair quality of life and can lead to serious complications including infertility, miscarriage, ectopic pregnancy, and chronic pelvic pain [1,2]. Before antibiotics, urethral stricture was a common sequela of gonorrhea in men [3]. Advanced and congenital syphilis continue to pose substantial burdens even in high-income countries, and untreated STIs increase the risk of HIV acquisition and transmission [4,5]. Consequently, STIs contribute to considerable global morbidity and economic cost. Incidence is strongly influenced by socioeconomic and behavioral factors [6].

The introduction of antimicrobials initially offered hope for effective STI treatment and complication reduction. In high-income countries, improved gonorrhea management has decreased pelvic inflammatory disease (PID) and, after a lag, ectopic pregnancy rates [7]. A major contemporary challenge is managing bacterial STIs detected through screening—testing asymptomatic individuals or those without known exposure. The situation is particularly alarming for *Neisseria gonorrhoeae*, where even last-resort empirical monotherapy (injectable ceftriaxone) is under threat, and for *Mycoplasma genitalium*, which has few treatment options and where untreatable infections have already been reported [8,9].

## 2 Epidemiology and clinical features

In 2020, the World Health Organization (WHO) estimated approximately 374 million new annual cases of four curable STIs—gonorrhea, chlamydia, syphilis, and trichomoniasis—among adults aged 15–49 years worldwide [6]. Although WHO does not provide estimates for *M. genitalium*, studies suggest its prevalence approaches that of chlamydia in examined populations [10]. Reliable population-level STI data are essential for accurate global and regional assessments of STI burden.

STI distribution varies widely by region and demographic group, influenced by sexual behavior, identity, geography, socioeconomic status, cultural factors (e.g., stigma, taboos), and access to sexual education, prevention, diagnostics, and healthcare investment [6,11].

## 3 Antimicrobial resistance and determinants

### 3.1 N. gonorrhoeae

Since antimicrobial therapy for gonorrhea was introduced in the 1930s, *N. gonorrhoeae* has repeatedly developed resistance through mutation or gene acquisition from other *Neisseria* species [11,12]. Over nine decades, it has become resistant to every previously used first-line monotherapy, including sulfonamides, penicillin, tetracyclines, spectinomycin, early macrolides, cephalosporins, and fluoroquinolones [12]. Resistance mechanisms include enzymatic inactivation, target modification, reduced uptake, and increased efflux [11,13]. Some determinants act independently, while others combine to produce clinical resistance [8] (Table 1). Genetic validation (e.g., transformation) rather than statistical correlation is essential for accurate resistance prediction [14].

**Table 1:** Molecular antimicrobial resistance determinants and their effects for antimicrobials against *Neisseria gonorrhoeae*.

Antimicrobial agent	Resistance determinant	Effect	Reference
Azithromycin	23S rRNA gene mutations: A2059G/A2058G (high-level resistance), C2611T (intermediate)	Reduced drug binding to 50S ribosomal subunit (peptidyl transferase loop in 23S rRNA domain V)	[66]
Ceftriaxone and cefixime	1. Mosaic *penA* alleles (e.g., PBP2 with A311V, I312M, V316 T/P, T483S, N512Y, G545S); *penA*-60 allele most prevalent 2. Non-mosaic *penA* with A501 V/T/P plus G542S and/or P551 S/L	Decreased acylation efficiency of PBP2 target, reducing cephalosporin susceptibility	[13,67]
Ciprofloxacin	1. GyrA mutations: S91F, D95 A/G/N2. ParC mutations: D86N, S87R/I/N, S88P, E91K	Impaired drug binding to DNA gyrase (GyrA) and topoisomerase IV (ParC)	[68]
Spectinomycin	1. 16S rRNA C1192T 2. RpsE mutations: T24P, V27del, K28E	1. Reduced drug affinity for 16S rRNA helix 34 2. Altered 30S ribosomal protein S5 (RpsE) binding site	[14]
Other antimicrobials	1. *mtrR* variants: promoter mutations (delA, A56C, mtr120), coding mutation G45D 2. *penB* mutations (PorB1b G120K/D, A121D)	1. MtrCDE efflux pump overexpression 2. Reduced porin-mediated drug influx	[14,69]

As ceftriaxone resistance and treatment failures emerged, dual therapy with ceftriaxone plus azithromycin was adopted [15,16]. However, rising azithromycin resistance has left ceftriaxone as the sole effective empiric agent. Ceftriaxone resistance and failures are now documented globally [8]. The first dual-therapy failure was reported in the UK in 2016 (originating in Japan) [17]. The multidrug-resistant FC428 clone, first identified in Japan in 2015 [18], has spread internationally and is endemic in Japan, Cambodia, and China [[19], [20], [21]]. Strains combining ceftriaxone and high-level azithromycin resistance were detected in England and Australia in 2018 (linked to Southeast Asia) [22,23] and in Austria in 2022 (linked to Cambodia) [23]. These typically carry the *penA*-60.001 mosaic allele, encoding a modified penicillin-binding protein 2 (PBP2) with reduced ceftriaxone affinity [19,24]. This allele is now predominant in ceftriaxone-resistant strains [21,25], including a recently identified resistant strain from the usually more susceptible lineage B [26], suggesting that ceftriaxone resistance can arise across the genetic diversity of *N. gonorrhoeae*.

Most ceftriaxone-resistant isolates have been linked to Asia, particularly the WHO Western Pacific and Southeast Asian regions, where antibiotic resistance is more prevalent [27]. Resistant strains often emerge in Asia before global spread, underscoring the need for robust, standardized international surveillance [27]. The WHO Global Gonococcal Antimicrobial Surveillance Program (GASP) collaborates with national programs to monitor resistance trends. To address GASP limitations (e.g., small samples, variable methods), the WHO Enhanced GASP (EGASP) incorporates test-of-cure assessments and whole-genome sequencing (WGS) where feasible [28].

WGS has transformed gonococcal epidemiology, enabling tracking of resistant and susceptible strains. Although applied regionally, its full potential is realized when combined with phenotypic and epidemiological data [27].

### 3.2 M. genitalium

Rising AMR in *M. genitalium* is a serious public health concern. Macrolide resistance is mainly driven by single-nucleotide polymorphisms (SNPs) in the 23S rRNA gene at positions A2058 or A2059 (*Escherichia coli* numbering) [29] (Table 2). Because *M. genitalium* has only one rRNA operon, a single mutation can raise the azithromycin minimum inhibitory concentration (MIC) from <0.063 mg/L to >8 mg/L [30]. High mycoplasmal mutation rates mean macrolide resistance mutations (MRMs) may arise spontaneously during untreated infection. Some evidence suggests that doxycycline-based resistance-guided sequential therapy reduces bacterial load and may lower MRM selection risk [31].

**Table 2:** Antimicrobial resistance determinants in *Mycoplasma genitalium*.

Resistance determinant	Fluoroquinolones	Tetracyclines	Macrolides
Major	*ParC*: S83I, S83R, D87N, D87Y *GyrA*: M95I, D99Y	No major determinants described	23S rRNA gene SNPs: A2058G, A2059G, A2058C, A2058T, A2059C
Minor	*ParC*: S83N, S83C, D87G, G81S *GyrA*: M95V, D99N	16S rRNA gene: GC966-967, C1192; ABC transporter gene mutations	A2062 (josamycin); A2058 with A2062 (pristinamycin); A2059T

Reported macrolide resistance rates vary geographically. Exceeding 50% in some sexual health clinic populations, likely reflecting higher azithromycin exposure, especially among men who have sex with men (MSM) [9]. Notably, Scandinavian countries show divergent patterns: Denmark and Norway report >55% MRM prevalence, whereas Sweden—where doxycycline is first-line for chlamydia and non-gonococcal urethritis (NGU)—has only 18% [32]. A proposed threshold of ∼1.3 defined daily macrolide doses per 1000 inhabitants per day may drive resistance above 5% [33]. A single 1 g azithromycin dose selects for resistance in 12–18% of cases, suggesting any macrolide use could exacerbate resistance [34,35].

Moxifloxacin resistance arises from SNPs in the *parC* quinolone resistance-determining region (QRDR), especially at S83 and D87 (*M. genitalium* numbering) [36] (Table 2). These QRDR mutations (QRAMs) are increasing globally [9], exceeding 80% in some Western Pacific regions [32]. Not all *parC* mutations (e.g., S83N) increase MICs or guarantee treatment failure [37]. The S83I mutation fails in ∼60% of moxifloxacin-treated cases, whereas wild-type *parC* predicts cure in 96% of patients, supporting its use in test-of-cure guidance [38,39]. Concurrent *gyrA* mutations (e.g., M95I, D99Y) further increase MICs and correlate with higher failure rates (81% vs. 43% for S83I alone).

Dual macrolide-fluoroquinolone resistance is now critical in many regions. With third-line therapies costly, scarce, and often ineffective, new treatments are urgently needed [40,41].

Zoliflodacin, a novel GyrB inhibitor, retains *in vitro* activity against dual-resistant strains, although a *gyrB* D443N mutation (MIC = 4 mg/L) was reported in one isolate [42]. Gepotidacin, another topoisomerase II inhibitor, shows potent bactericidal effects *in vitro*, including against resistant strains, though clinical data are limited [43].

Despite doxycycline's high clinical failure rate (60–70%), *in vitro* resistance is rare (MIC_90_ = 1 mg/L), and MICs do not predict outcomes [44]. Tetracyclines bind 16S rRNA helices 31 and 34; in *Mycoplasma bovis*, SNPs at A965, A967, or G1058 (*E. coli* numbering) confer resistance (Table 2). Although similar SNPs occur in *M. genitalium*, no clinical correlation has been established [45]. Prolonged *in vitro* doxycycline exposure did not induce resistance, though H31 mutations appeared in 5% of clinical samples. A single tetracycline-resistant isolate (MIC = 32 mg/L) from an immunocompromised patient had an ABC transporter mutation but no *tetM* gene [46,47].

Resistance in *M. genitalium* appears polyclonal, likely due to ease of SNP development. Genomic studies are challenging due to fastidious growth requirements, often requiring Vero cell culture or direct sequencing of clinical specimens, which is complicated by host DNA contamination [48]. Phylogenetic analysis of 28 axenic isolates revealed two major clades without clear geographic or AMR associations. Notably, five presumed unique strains in the ATCC collection were identical to the G37 reference strain, suggesting cross-contamination and raising questions about prior AMR data derived from them [49].

## 4 Discussion

The growing AMR threat poses significant treatment challenges for *N. gonorrhoeae* and *M. genitalium*. In contrast, *Chlamydia trachomatis* and *Treponema pallidum* remain fully susceptible to first-line therapies [50,51]. These latter pathogens are primary targets of doxycycline post-exposure prophylaxis (doxy-PEP), where individuals take 200 mg doxycycline within 24–72 h after unprotected sex. Trials in France and the United States (US) showed significant reductions in chlamydia and syphilis incidence, with additional reductions in gonorrhea in the US study [51].

Concerns remain about doxy-PEP potentially selecting for tetracycline resistance in *C. trachomatis* and *T. pallidum*, especially given global prevalence of tetracycline-resistant *N. gonorrhoeae* [[52], [53], [54]]. Other concerns include doxycycline's role in treating NGU and *M. genitalium*, and potential impacts on human microbiomes and broader AMR patterns [54].

With ceftriaxone resistance rising, the gonorrhea treatment arsenal is dangerously limited. Several candidates (e.g., delafloxacin, solithromycin) failed phase III trials [55,56], leaving zoliflodacin and gepotidacin as the most advanced alternatives [57,58]. Zoliflodacin showed 100% efficacy for urogenital/rectal infections (78% pharyngeal) in phase II, and recently met non-inferiority criteria in phase III versus ceftriaxone/azithromycin combination therapy [59]. However, *in vitro* studies identified zoliflodacin-resistant mutants with GyrB mutations (D429 or K450) increasing MICs to 1–2 mg/L [60]. Gepotidacin demonstrated 95–97% efficacy in phase II for urogenital gonorrhea, with failures associated with pre-existing ParC D86N mutations and emergent GyrA A92T substitutions (MICs ≥32 mg/L) [61]. Phase III results are expected in 2025. Lefamulin shows *in vitro* promise but has suboptimal pharmacokinetics for gonorrhea and lacks clinical trial data. Other investigational compounds (novel tetracyclines, carbapenems, quinolones) show adequate MICs but lack clinical evidence [62,63].

Current third-line options for *M. genitalium* (pristinamycin, sitafloxacin, minocycline) are limited by availability, cost, and suboptimal efficacy. Although gepotidacin, zoliflodacin, and lefamulin demonstrate *in vitro* activity against resistant strains, clinical performance has been disappointing, especially for lefamulin, which showed poor MIC–outcome correlation [64]. This parallels the doxycycline paradox, where *in vitro* susceptibility contrasts with high clinical failure rates [44,64].

Emerging data suggest potential roles for nitroimidazoles (e.g., metronidazole in PID) and chloramphenicol/thiamphenicol, though more research is needed. New tetracycline analogs (omadacycline, eravacycline) show *in vitro* promise but have been tested on limited isolates [65].

## 5 Conclusion

The AMR crisis in STIs, particularly gonorrhea and *M. genitalium*, requires coordinated global action. Enhanced surveillance, resistance-guided therapy, rapid diagnostics, and investment in novel therapeutics are imperative. While new agents like zoliflodacin and gepotidacin offer hope, pre-existing and emergent resistance threaten their long-term utility. Public health strategies must integrate antimicrobial stewardship, targeted prophylaxis, and ongoing research to prevent a return to untreatable STIs.

## CRediT authorship contribution statement

**Nafiseh Ghodsi:** Writing – review & editing, Writing – original draft, Validation, Data curation. **Roghayeh Arezoomand:** Writing – original draft, Validation, Software, Resources. **Amir Azimian:** Writing – review & editing, Writing – original draft, Visualization, Validation, Supervision, Software, Resources, Project administration, Methodology, Investigation, Funding acquisition, Formal analysis, Data curation, Conceptualization.

## Ethical approval

Not applicable.

## Funding

This work was supported by project number IR.NKUMS.REC.1402.164.

## Declaration of competing interest

The authors declare that they have no known competing financial interests or personal relationships that could have appeared to influence the work reported in this paper.
